# Quantitative phosphoproteomics reveals GSK3A substrate network is involved in the cryodamage of sperm motility

**DOI:** 10.1042/BSR20211326

**Published:** 2021-10-12

**Authors:** Jing Wang, Jing Wang, Min Wang, Renyun Hong, Shanshan Tang, Yuanhua Xu, Xia Zhao, Tao Zhou, Zibin Wang, Shaoping Huang

**Affiliations:** 1 Department of Reproductive Medicine, Zhongda Hospital, School of Medicine, Southeast University, Nanjing 210009, China; 2 Research Institute for Reproductive Medicine and Genetic Diseases, The Affiliated Wuxi Maternity and Child Health Care Hospital of Nanjing Medical University, Wuxi 214002, China; 3 Analytical and Testing Center, Nanjing Medical University, Nanjing 211166, China; 4 Department of Human Anatomy and Neuroscience, Medical School, Southeast University, Nanjing 210009, China

**Keywords:** cryodamage, GSK3A, kinase activity, phosphorylation, sperm cryopreservation, sperm motility

## Abstract

During sperm cryopreservation, the most significant phenotype of cryodamage is the decrease in sperm motility. Several proteomics studies have already been performed to search for key regulators at the protein level. However, sperm functions are known to be highly regulated by phosphorylation signaling. Here, we constructed a quantitative phosphoproteome to investigate the expression change of phosphorylated sites during sperm cryopreservation. A total of 3107 phosphorylated sites are identified and 848 of them are found to be significantly differentially expressed (DE). Bioinformatics analysis showed that the corresponding genes of these regulated sites are highly associated with sperm motility, providing a connection between the molecular basis and the phenotype of cryodamage. We then performed kinase enrichment analysis and successfully identified glycogen synthase kinase-3α (GSK3A) as the key kinase that may play an important role in the regulation of sperm motility. We further constructed a GSK3A centric network that could help us better understand the molecular mechanism of cryodamage in sperm motility. Finally, we also verified that GSK3A was abnormally activated during this process. The presented phosphoproteome and functional associations provide abundant research resources for us to learn the regulation of sperm functions, as well as to optimize the cryoprotectant for sperm cryopreservation.

## Introduction

Sperm cryopreservation via liquid nitrogen is a valuable technology for male fertility preservation. It was widely applied in clinical cases such as long-term storage for sperm donation, sperm preservation before cancer therapy or vasoligation, and sperm preparation for severe oligoathenospermia. Although the pregnancy probability of frozen sperm could last for several decades of years [1], it is clear that the process of freezing and thawing is harmful for human sperm. Compared with fresh sperm, freeze-thawing may have various side effects on DNA integrity, membrane morphology, motility, and viability [2,3]. The most significant phenotype of cryodamage is the decrease in sperm motility. The reductions in sperm motility are found to be between 25 and 75% [4]. Low sperm motility is also highly associated with male infertility. A previous study based on large population reveals that approx. 81.84% of the infertile men had impaired sperm motility [5]. Thus, it is important to study the corresponding molecular changes during the process of sperm cryopreservation and thawing, which may help us to understand the mechanism of cryodamage or to improve the technology of cryopreservation. In addition, it may also provide novel hints for the diagnosis and treatment of asthenozoospermia.

Over the past decades, high-throughput proteomics technology based on liquid chromatography-tandem mass spectrometry (LC-MS/MS) has been fast developing and offers a great convenience to discover the whole protein composition in sperm samples [6]. By comparing different pathological or physiological conditions using label-free or labeling technologies, quantitative proteomics can identify key functional proteins or potential disease biomarkers [7]. For example, based on Tandem Mass Tag (TMT) labeling, a previous quantitative proteomics study identified differentially expressed (DE) proteins between sperm samples with different levels of motility [8]. These proteins were annotated to be highly associated with mitochondria-related energetic metabolic pathways. Recently, there are also several proteomics studies performed to investigate the molecular differences between fresh and cryopreserved (post-thaw) human sperms [9–12]. Interestingly, sperm proteins were found to be dynamically and remarkably changed during the complex procedures of adding cryoprotectant, cryopreservation, and thawing. Although the precise mechanism of cryoinjury is still not well-elucidated, several proteins associated with mitochondrial oxidative damage seem to play leading roles in this process. However, one of the drawbacks of these proteomics studies is that protein changes were only investigated at the overall expression level. We previously showed that sperm proteins with post-translational modifications (PTM)s, such as acetylation or phosphorylation, are more directly involved in sperm-specific functions [13]. Protein kinases (such as protein kinase A and B) and the corresponding phosphorylation-dependent signaling cascades are known to be crucial for the regulation of sperm motility [14]. Thus, it is of vital importance and necessity to discover the dynamic changes of protein phosphorylation during sperm cryopreservation.

It is known that most of the phosphopeptides are hard to be detected because of their low stoichiometry in the sample. Thus, phosphoproteomics analysis usually applies various strategies to enrich phosphopeptides for a more comprehensive profiling of protein phosphorylation [15]. We previously identified over 200 DE phosphorylated sites during the process of sperm capacitation [16]. Based on an enrichment analysis of kinase–substrate relations, we successfully identified a key tyrosine kinase (insulin growth factor 1 receptor) that may be essential for human sperm capacitation. Thus, quantitative phosphoproteomics is efficient in identifying both the phosphorylated sites and the upstream regulatory kinases. In the present study, we aim to investigate whether there is a connection between protein phosphorylation and the declining of sperm motility during cryopreservation. We will apply quantitative phosphoproteomics approach to systematically measure the expression changes of specific phosphorylated sites during this process. In addition, we also intend to predict the key upstream kinase and verify its activity. The present study could help us better understand the molecular mechanism of cryoinjury, as well as to provide novel insights and targets for the optimization of sperm cryopreservation.

## Materials and methods

### Ethical statement and sample collection

This project was a non-invasive investigation for the participants and was approved by the Ethics Committee of Southeast University. Prior to sample collection, written informed consents were obtained from all enrolled volunteers (recruited from local residents in Nanjing). All clinical information (including basic information and testing results) from volunteers is anonymized. As a basic research, the current results do not provide any health guidance or feedback to the participants.

Semen samples were obtained by masturbation into sterile containers after 3–5 days of abstinence. Only normal samples were used in the present study, as assessed based on semen analysis. A total of 30 samples were collected and randomly divided into three biological replications. Each replicate was further equally divided into four parts, which are used for labeling-based quantitative proteomics and phosphoproteomics analyses of fresh and freeze-thawed samples correspondingly.

Semen analysis was performed with a computer-assisted semen analysis (CASA) system. Normal semen quality was assessed according to the criteria of World Health Organization (WHO) 2010. Focused on sperm motility, the proportion of progressively motile sperms should be larger than 32%. The protamine-to-histone transition is an important indication for sperm maturation and function [17]. Thus, the protamine-to-histone ratios are also required to be in normal range based on the test of aniline blue staining. Additionally, to avoid possible effects of heavy metal pollution on sperm functions [18,19], we also performed trace elements (including copper and zinc) detection for the selection of normal samples.

### Sperm cryopreservation

The fresh ejaculated semen samples were first allowed to be completely liquefied at room temperature (25°C). Then, the samples were mixed with an equal volume of cryoprotectant (sperm freezing medium, Origio, Medicult, Denmark). Next, the equilibrated samples were divided into two aliquots. One part was used directly as control group. And the other aliquot was stored in liquid nitrogen for 7 days after a process of gradient cooling according to the manual of cryoprotectant. The semen samples were thawed by water bath at 37°C for 5 min. Finally, the samples were used as experimental group after a further equilibration period of 10 min at room temperature (25°C).

### Protein extraction, peptide labeling, and phosphopeptide enrichment

First, the collected samples were processed by centrifugation (2000×***g*** for 5 min) in a 60% Percoll gradient (GE Healthcare, Waukesha, WI, U.S.A.) to remove seminal plasma, immature germ cells, and non-sperm cells (mainly epithelial cells). Then, the purified sperm cells were further thrice washed in phosphate-buffered saline (PBS; pH 7.2) at 2000×***g*** for 5 min to further remove cryoprotectant. Next, sperm samples were lysed in 8 M urea lysis buffer with a cocktail of proteases’ inhibitors (Pierce, Rockford, U.S.A.). The lysates were centrifuged at 40000×***g*** for 60 min (4°C) following sonication for 10 s to extract protein component. Protein concentration was assessed using the Bradford method. Proportionally, the liquid with 2 mg proteins was reduced in 20 μl of 1 M dithiothreitol (DTT) at 56°C for 1 h to reduce disulfide bonds, and then treated with 100 μl of 1 M iodoacetamide (IAA) in the dark for 45 min for alkylation. After that, seven-times of the total liquid volume of acetone solution buffer were added overnight at −20°C to precipitate proteins.

Second, the protein samples were digested by trypsin at 37°C overnight subsequently quenched by addition of trifluoroacetic acid (TFA). The peptides were desalted using a SepPak column (Waters Co., Ltd, Milford, MA, U.S.A.). The desalted peptides were also divided into two parts for proteomics and phosphoproteomics analyses, respectively. For phosphoproteomics analysis, the peptides were further needed to enrich phosphopeptides (with serine, threonine, or tyrosine phosphorylated sites) by a combination of immobilized metal affinity chromatography (IMAC) beads and TiO_2_ beads as described previously [16].

For, peptide or phosphopeptide labeling was performed by using the TMT 6-plex isotopic label reagent (Thermo Scientific, Rockford, U.S.A.) according to the manufacturer’s instructions. The control groups (fresh sperm with cryoprotectant) were labeled with TMT-126, TMT-127, and TMT-128, while the experimental groups (thawed sperm) were labeled with TMT-129, TMT-130, and TMT-131, respectively. Finally, all six aliquots were combined for the following mass spectrometry analysis.

### Tandem mass spectrometry analysis

The peptide mixture was analyzed using an LTQ OrbitrapVelos mass spectrometer (Thermo Fisher Scientific, San Jose, CA) coupled directly to an LC system. The trap column effluent was transferred to a reverse-phase microcapillary column (0.075 × 150 mm, Acclaim PepMap100 C18 column, 3 μm, 100 Å; Thermo Fisher Scientific). The separation of peptides was performed using buffer A (2% acetonitrile and 0.5% acetic acid) and buffer B (80% acetonitrile and 0.5% acetic acid) under a 120-min gradient.

MS analysis was performed in data-dependent acquisition mode. An MS survey scan was obtained for the m/z (mass-to-charge ratios) range 350–1800 at a resolution of 60000. An MS/MS scan of every precursor in the octopole collision cell (higher energy collision dissociation, HCD) was acquired from the survey scan for the 20 most intense ions (as determined by Xcalibur mass spectrometer software in real time). Dynamic mass exclusion windows of 60 s were used, with siloxane (m/z = 445.120025) as a lock mass.

### Proteomics data analysis

Raw files were searched against the human proteome sequences obtained from the Universal Protein Resource (UniProt) database (release 202102) [20] using the MaxQuant software (version 1.5.2.8) [21]. False discovery rates (FDRs) were estimated using the target-decoy strategy. The FDR cutoff was set to 0.05 for sites, peptides, and proteins. Enzyme specificity was considered to be fully cleaved by trypsin, and two maximum missed cleavage sites were permitted. The minimum required peptide length was set to six residues. Carbamidomethylation of cysteine was set as fixed modification. Oxidation of methionine and phosphorylation (serine, threonine, or tyrosine) were considered as variable modifications. The mass tolerance for precursor ions was set to 6 ppm. The mass tolerance for fragment ions was set to 0.5 Da.

Quantification of protein groups or phosphorylated sites was based on the reporter ion intensities of TMT6 reagents at the peptide level [22]. The quantification results by MaxQuant were further optimized using the automatic reporting tool, MaxReport (version: 2.2) [23]. The relative expression values for every protein groups and sites among different samples were calculated by a modified Libra algorithm embedded in MaxReport. The expression differences between control and experimental groups were calculated using the Student’s *t* test. For multiple testing correction, the FDR was further calculated by using the Benjamini–Hochberg method. It is known that the expression changes of quantitative proteomics based on labeled reagents are usually underestimated due to its intrinsic features [24]. Thus, the criteria for screening of statistically DE proteins or sites were based on a combination of FDR (less than 0.05) and absolute fold change (larger than 1.5). To further visualize the results of expression analysis, volcano scatter and circular heatmap were used to display the overall and differentially expression results correspondingly. The volcano scatter plot shows the distribution of each protein or site according to its expression change (x-axis for log2 transformed fold change) and statistical significance (−log10 transformed FDR). Thus, the upper-left and upper-right regions (as clearly distinguished by the threshold lines of fold change and FDR) of the plot indicates the statistically DE proteins or sites. The circular heatmap specifically displays the clustered expression values, fold changes, and FDR values of DE proteins or sites in circular tracks. All the graphs were plotted by using the R software (version: 4.0.2).

### Bioinformatics analysis

The ToppGene web tool (version: 2021.3) [25] was applied for gene functional classification and enrichment analyses based on annotations from the Gene ontology (GO) [26] and the Mammalian Phenotype (MP) [27] databases. For the convenience of functional annotations, the DE sites or protein IDs were mapped into Ensemble genes. The whole human genome was set as background and an adjusted *P*-value (by the Benjamini and Hochberg method) of less than 0.05 was considered statistically significant. The STRING database (version: 11.0) [28] was used for analyzing the interacting relations among a list of proteins. The cytoscape software (version: 3.8.2) [29] was further used to refine and construct complex network between proteins and functions.

For phosphorylation specific data analysis, the iGPS software (version: 1.0.1) [30] was used to predict kinase–substrate relationships. Enrichment analysis based on Fisher’s exact test was also performed to identify the dominant regulatory kinases.

### Transmission electron microscopy analysis

For transmission electron microscopy analysis, samples were fixed with 2.5% glutaraldehyde buffered with 0.1 M sodium phosphate, pH 7.4. Then, the samples were further post-fixed for 2 h with 1% osmium tetroxide, dehydrated in ethanol, embedded in Epon812, and polymerized at 60°C for 48 h. After ultrathin sectioning (Leica, UC7) and staining with 2% uranyl acetate and lead citrate, the samples were finally observed in a transmission electron microscope (FEI, Tecnai Spirit Biotwin).

### Label free-based verification of phosphorylated site

To verify the existence and expression change of phosphorylated site, a total of ten independent samples were additionally collected and processed as described above. The equilibrated samples (with cryoprotectant) were also divided into two groups: control and experimental (post-thaw) groups. The following steps of protein extraction, digestion, and LC-MS/MS analyses were the same with the above, except for peptide labeling and mixture. The detailed method of label-free was described previously [16].

## Results and discussion

### Comparison of effects on sperm parameters during sperm cryopreservation

**Figure 1:**
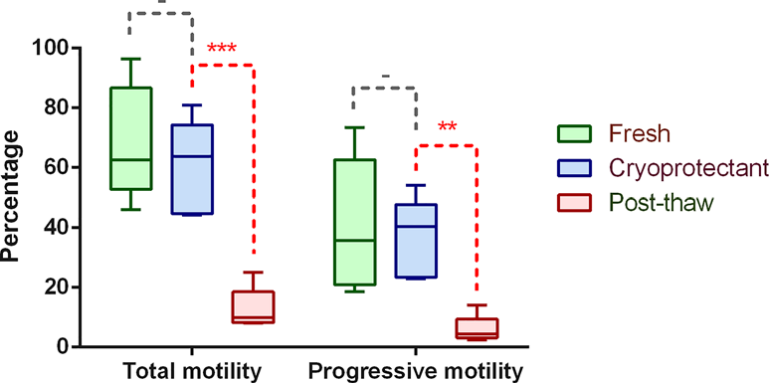
Comparison of sperm parameters during sperm cryopreservation The paired Student’s *t* test was used for comparisons between different groups. Statistical significance: - (*P*>0.05), ** (*P*<0.01), *** (*P*<0.001).

During the process of freezing and thawing, there are actually two main factors (including cryoprotectant and cryodamage) that may affect sperm parameters. We thus compared the influences of these two factors based on the results of semen analysis. As shown in Figure 1, the total motility and progressive motility are slightly decreased after the addition of cryoprotectant, although not statistically significant. However, the two parameters are dramatically decreased after thawing. Additionally, we also checked the morphological features by light microscopy (using Diff-Quick staining method) and found no obvious alterations during each step (Supplementary Figure S1). These results are consistent with previous study based on large dataset and also verify that the impaired sperm motility is the main phenotype of cryodamage [4].

### Quantitative phosphoproteomics analysis of human sperm during cryopreservation

**Figure 2:**
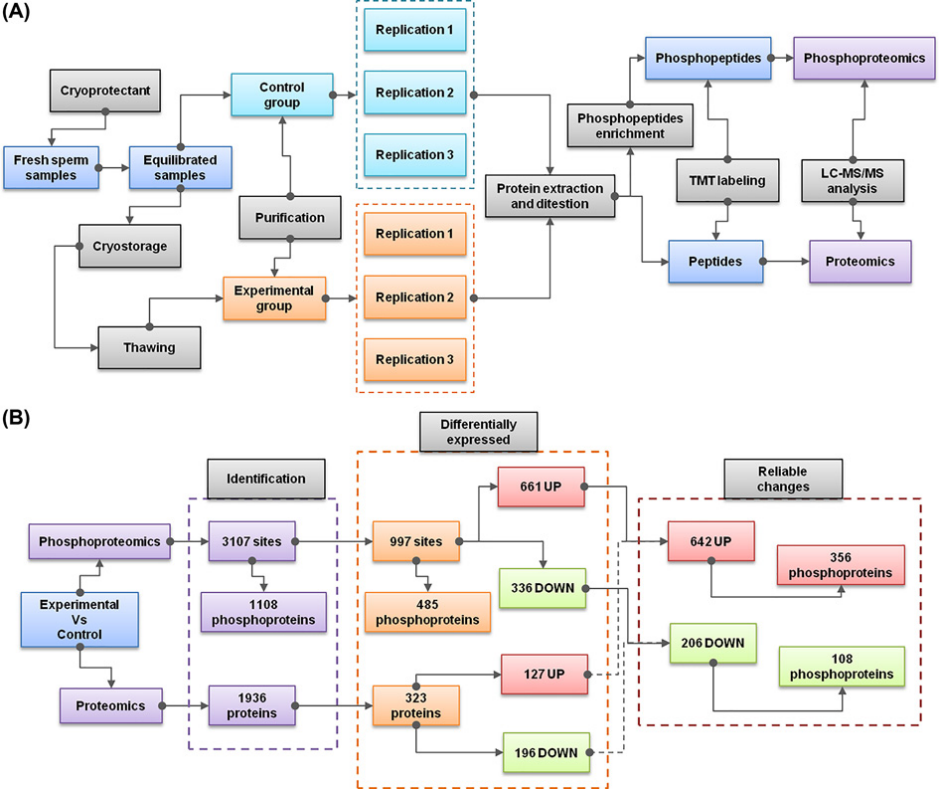
Schematic diagrams of the experimental design and summarized results (**A**) The overall experimental design including sample grouping, sample processing, phosphoproteomics procedure, and proteomic procedure. (**B**) Summary of the identification and statistical results of phosphoproteomics and proteomics analysis.

Previously, there are four representative proteomic studies, which have already investigated the global protein changes based on two-dimensional gel electrophoresis (2DE) [9], TMT labeling [10], isobaric tags for relative and absolute quantitation (iTRAQ) [11], or data-independent acquisition mass spectrometry (DIA-MS) [12] separately. However, PTMs such as phosphorylation are known to play key roles in regulating sperm functions. Currently, there is lack of published studies, which have comprehensively investigated this issue. Thus, our work mainly focuses on the phosphorylated proteins and the corresponding sites of human sperm proteins. Since the addition of cryoprotectant has few effects on sperm motility, most of previous proteomics studies only compared fresh sperms with thawed sperms [9,11,12]. Only one study specifically collected sperm samples with cryoprotective regents as the control group [10]. The aim of our study is to discover the molecular changes of cryodamage. Thus, it is more accurate to treat equilibrated sperm samples with cryoprotectant as the control group (Figure 2A). While the thawed sperm samples were selected as experimental group. Each group was divided into three replications. After protein extraction and digestion, phosphopeptides were enriched and labeled for quantitative phosphoproteomic analysis. In the meantime, we also construct a proteomic profiling of the overall protein expression by using the same samples.

In summary, a total of 3107 phosphorylated sites are identified in the phosphoproteome, corresponding to 1108 phosphoproteins (Figure 2B; Supplementary Data S1). Using a combined criteria of FDR (less than 0.05) and absolute fold change (larger than 1.5), 661 up-regulated (experimental versus control) sites and 336 down-regulated sites were found to be DE significantly. We further removed those DE sites that may caused by the changes of their corresponding protein levels, according to the matched results of the whole proteome. Finally, we obtained a reliable list of DE sites, including 642 up-regulated sites (corresponding to 356 phosphoproteins) and 206 down-regulated sites (corresponding to 108 phosphoproteins).

**Figure 3:**
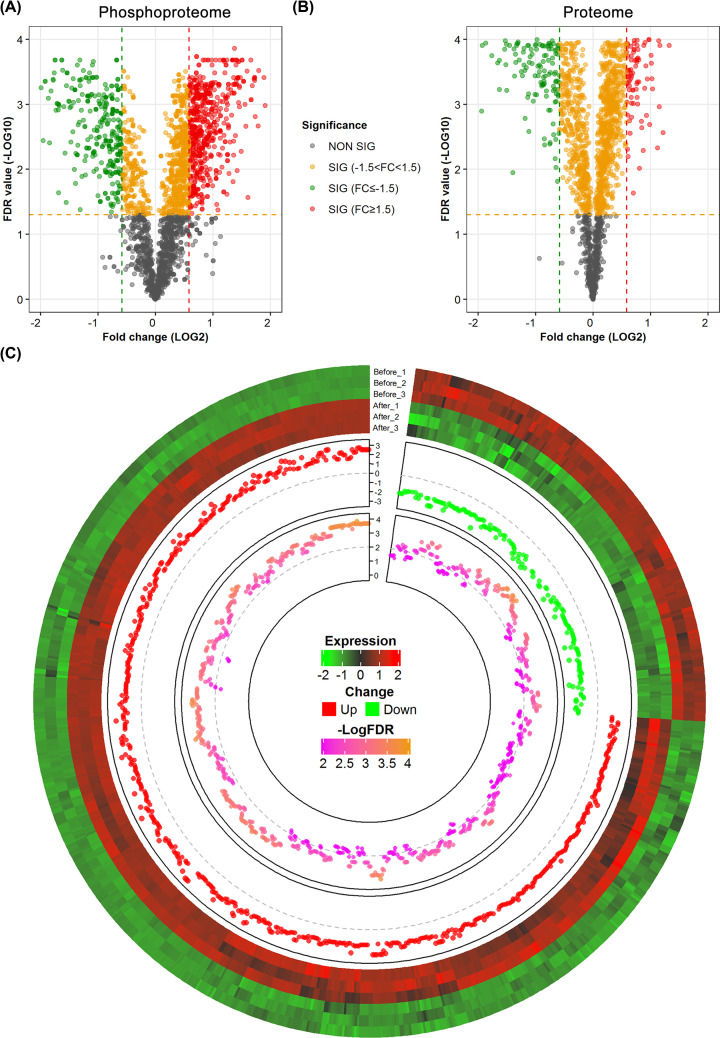
Expression analysis of the phosphoproteome and proteome (**A**) Volcano plot of the whole phosphoproteome. The x-axis represents fold change (log2 transformed), while the y-axis indicates the statistical significance (−log10 transformation of FDR values). (**B**) Volcano plot of the whole proteome. Each site of protein is classified according to its values of fold change and statistical significance. (**C**) Circular plot of the reliable list of DE sites. From outer to inner, the track represents expression clustering, fold change, and significance (log-transformed FDR), respectively.

Both the overall expression of phosphoproteome and proteome showed a distinct expression pattern between the control (before cryostorage) and thawed groups (Supplementary Figure S2). However, most of the phosphorylated sites tend to be up-regulated after thawing, especially for DE sites (Figure 3). Among the list of reliable DE sites, up-regulated sites are approx. three times as much as the down-regulated sites. On the contrary, only a slightly more proportion of down-regulated proteins are found in the whole proteome. Interestingly, the degree of expression change is also seen to be more dramatic in phosphorylated sites, compared with protein levels. In addition, although more than a half of the phosphoproteins are quantified in the proteome, only approx. 5% of the phosphoproteins with DE sites are overlapped with the DE proteins in the proteome (Supplementary Figure S3). It indicates that most of the changes of phosphorylated sites are highly regulated during the process of cryopreservation, and are not caused by the expression changes of their corresponding proteins. Combing the above results, it is more important to study the phosphorylated sites to search for key targets for understanding the molecular mechanism of cryodamage.

In addition, it also should be noted that the present study only focused on O-linked phosphorylation (including serine, threonine, or tyrosine sites), which commonly occur in eukaryotes. Currently, there lacks efficient methods for the protection and enrichment of the unstable N-linked phosphorylation (including histidine, arginine, and lysine), which are also usually low abundance [31]. However, previous studies showed that the histone-to-protamine transition and lysine-to-arginine ratios are highly associated with sperm maturation, motility, and DNA integrity [17,32]. Thus, it is necessary and interesting to complementary study O-linked phosphorylation during this process in the future.

### Functional enrichment analysis of DE phosphorylated sites

**Figure 4:**
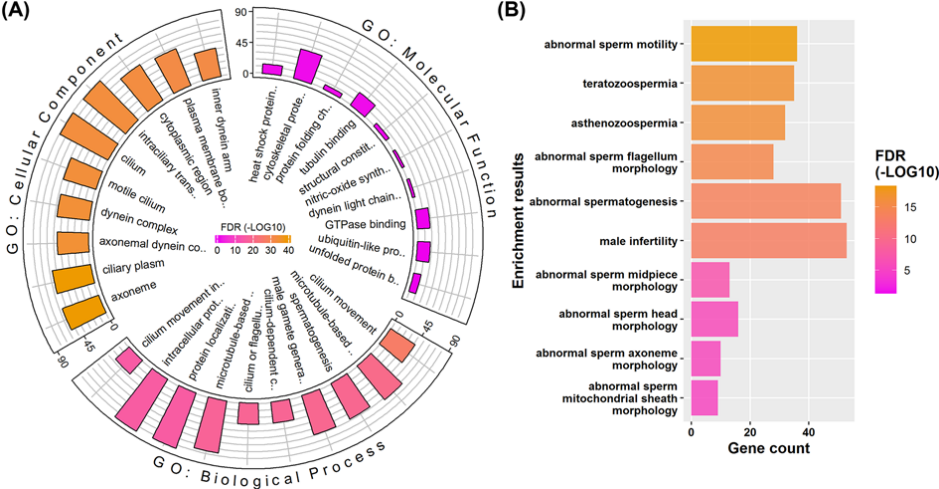
Representative enrichments of DE phosphorylated sites at gene level (**A**) GO enrichment results. (**B**) MP enrichment results. The scaled colors are corresponding to the significance of FDR values (log-transformed). The bar plot indicates the number of genes.

Functional enrichment analysis could help us better organize and prioritize the results of omics-based data. The GO is one of the most widely used functional databases. It provides gene annotations at three hierarchical layers: biological process, cellular component, and molecular function. The reliable DE phosphorylated sites are mapped to 477 genes. Functional enrichment analysis shows that these genes are mainly involved in cilium- or flagellum-related movement in terms of biological process (Figure 4A; Supplementary Data S2). Consistently, these genes are prone to be located in the cellular parts such as cilium or axoneme. We further performed transmission electron microscopy analysis to compare the ultrastructural changes during this process. Consistent with the annotations, there are obvious structural abnormalities (broken or twisted axoneme) in the midpiece of the thawed sperm (Supplementary Figure S4). For molecular functions, most of the enriched binding activities are also related to microtubule motor. These results show that there may be a strong connection between the expression changes of phosphorylated sits to the main phenotype of cryodamage (decrease in sperm movement) during sperm cryopreservation.

Since human genes are hard to be directly studied *in vivo*, there is a lack of enough annotations for male-specific phenotypes. Mouse is the most studied animal model for us to understand mammalian gene function. Thus, homologous genes and their associated phenotypes as annotated in the MP databases can be used to improve human gene annotation, especially for disease analysis [33]. Among the genes associated with DE sites, we found that many diseases or phenotypes that directly associated with sperm functions are statistically enriched (Figure 4B; Supplementary Data S2). As expected, the most significantly enriched phenotype is ‘abnormal sperm motility’. Specifically, these genes are highly associated with abnormal morphology of sperm head, midpiece, mitochondrial sheath, axoneme, and flagellum. First, these results further verify the relationship between the expression changes of phosphorylated sites and the decrease in sperm motility. Second, the results also provide a candidate list of targets, which could be used to explain the decrease in sperm motility at PTM level.

Additionally, we also performed GO and MP enrichment analyses using the DE proteins identified in the proteome. Interestingly, many of enriched terms are the same with the results of DE sites. However, the FDR values of enrichment are generally lower than those of DE sites, and the corresponding numbers of key genes are also smaller. For example, a total of 36 genes of the DE sites are associated with abnormal sperm motility, while the corresponding gene number of DE proteins is only 19 (Supplementary Figure S5). Since the gene compositions of DE proteins and DE sites are largely different, the expression turnover at both protein and site levels may contribute to the decrease of sperm motility during cryopreservation. We believe that phosphorylation may play a more important role in this process, since DE sites are more strongly correlated with sperm motility.

### Prediction of upstream regulatory kinases

**Figure 5:**
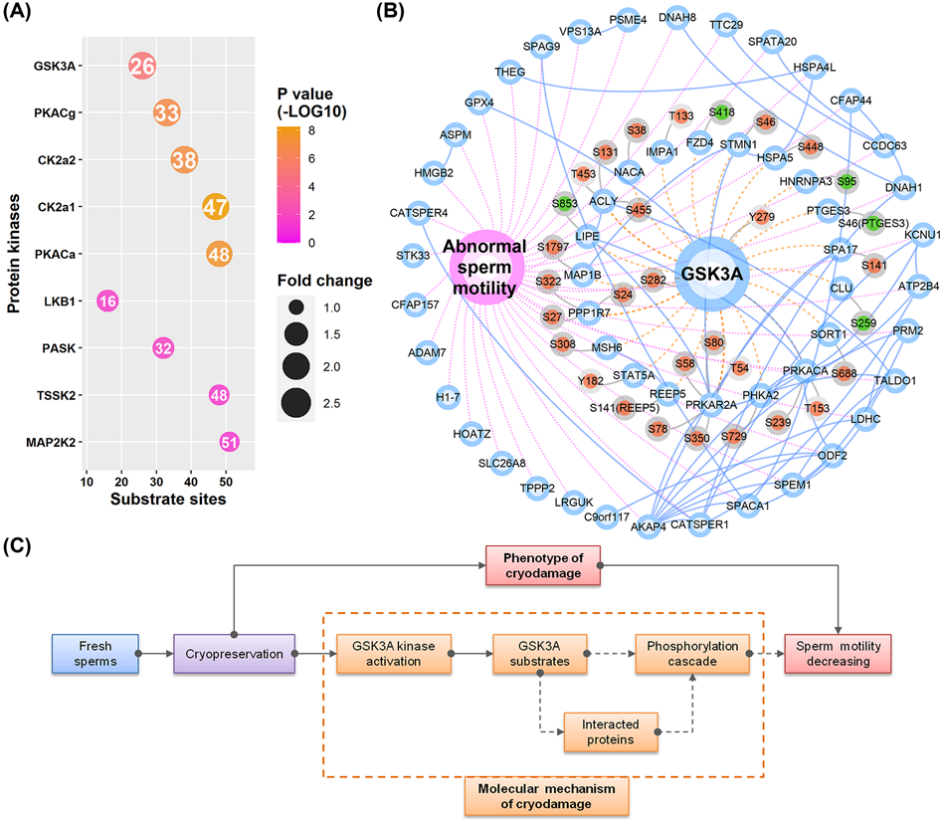
Kinase enrichment and GSK3A centric kinase–substrate network (**A**) The enriched list of kinases, sorted by fold change in reverse order. The scaled colors indicate the *P*-values (log-transformed). The size of the dot is proportional to fold change. Circles with purple, blue, and gray borders represent phenotype, proteins, and sites, respectively. Red and blue colors for the sites represent up-regulated and down-regulated expression, respectively. The number in the dot shows the count of substrate sites. (**B**) GSK3A centric kinase–substrate network. Blue solid lines, purple dashed lines, brown dashed lines, and gray solid lines indicate protein-to-protein interactions, gene-to-function relations, kinase–substrate relations, and protein–sites relations, respectively. (**C**) A schematic diagram for the connection between GSK3A kinase signaling and the decrease in sperm motility. Abbreviation: GSK3A, glycogen synthase kinase-3α.

Besides the identification of phosphorylated sites, phosphoproteomics data can also be used to effectively screen upstream regulatory kinases. It is known that the binding of kinases and substrates is highly selective and rely on the recognition of specific motifs [34]. We previously successfully identified key regulatory kinases for mouse spermatogenesis [35], mouse spermatogonial progenitor cell proliferation [36], and human sperm capacitation [16], based on an improved bioinformatics enrichment method. Here, we also applied similar method to search for potential kinases that may be involved in regulating these DE sites. In brief, we first predicted all possible paired site-specific kinase–substrate relations based on all identified sites using the iGPS software. To obtain reliable results, we only considered those kinases that are identified in the current phosphoproteome or protoeme. Then, a Fisher’s exact test was used to identify ‘active’ or ‘inhibited’ kinases by comparing the ratios of DE substrate sites. Among the 27 identified kinases, 9 were significantly enriched up-regulated DE substrate sites (Figure 5A; Supplementary Data S3). Interestingly, all significant kinases are classified as ‘active’ kinases. The results are consistent with the expression change of DE sites, as most of them are up-regulated sites.

We further applied multiple criteria to prioritize these kinases: absolute fold change > 1.5, *P*-value <0.001, substrate genes are functionally associated with sperm motility, and evidences for the change of kinase activity. Finally, we focused on glycogen synthase kinase-3α (GSK3A) and speculated that it may play the most important role in the cryodamage of sperm motility. Considering the values of fold change, GSK3A is the most significantly changed with an enrichment fold of 2.06. It also has three substrate genes, including GSK3A, PRKACA (cAMP-dependent protein kinase catalytic subunit α) and LIPE, which are known to be directly involved in regulating sperm motility. Other kinases such as PKACg, may have some common substrate sites with GSK3A. However, GSK3A is the only enriched kinase that is shown to be activated in the thawed sperm samples. The kinase activity of GSK3A is determined by the phosphorylation Y279 (tyrosine at position 279). In the phosphoproteome, we found that the phosphorylation level of Y279 is significantly up-regulated in the thawed sperm samples, indicating GSK3A is abnormally activated during the process of sperm cryopreservation.

There are a total of 31 DE substrate sites for GSK3A (Table 1), including 26 up-regulated sites (corresponding to 16 genes) and 5 down-regulated sites (corresponding to 5 genes). Only three substrate genes (including GSK3A, PRKACA, and LIPE) as mentioned above are known to be directly associated with sperm motility. However, considering the interacting proteins of GSK3A and its substrates, there are 18 more substrate genes (including AKAP4, ATP2B4, CATSPER1, CATSPER4, CFAP44, DNAH1, DNAH8, GPX4, HSPA4L, KCNU1, LDHC, ODF2, PRM2, SPACA1, SPAG9, SPEM1, TALDO1, and THEG) that could be indirectly connected to GSK3A and sperm motility (Figure 5B). In other words, among the total 36 genes (derived from the DE sites) annotated to be involved in sperm motility, approx. 58% (21 genes) of them are also directly or indirectly associated with GSK3A.

**Table 1:** Differentially expressed substrate sites of GSK3A

Protein ID	Gene name	Position	Modified residue	Fold change	Significance
P53396	ACLY	453	T	1.96	***
P53396	ACLY	455	S	1.96	***
Q15506	SPA17	141	S	1.54	***
P13861	PRKAR2A	78	S	1.64	***
P13861	PRKAR2A	80	S	1.64	***
K7ERP6	PRKACA	239	S	1.93	***
P49840	GSK3A	282	S	1.88	***
P49840	GSK3A	279	Y	1.88	***
P13861	PRKAR2A	54	T	1.52	***
P13861	PRKAR2A	350	S	1.64	***
Q15435	PPP1R7	27	S	1.59	***
Q15435	PPP1R7	24	S	1.59	***
E5RG13	IMPA1	133	T	2.09	***
A2A2D0	STMN1	46	S	1.53	***
P13861	PRKAR2A	58	S	1.56	***
E2QRG8	REEP5	141	S	1.70	***
P11021	HSPA5	448	S	1.68	***
Q15435	PPP1R7	322	S	1.59	***
F8W1N5	NACA	38	S	1.54	**
Q99523-2	SORT1	688	S	1.55	**
P46821	MAP1B	1797	S	1.52	**
A0A087WWJ1	MSH6	308	S	2.81	**
K7EM17	STAT5A	182	Y	1.57	**
K7EIE7	ACLY	131	S	1.93	**
K7ERP6	PRKACA	153	T	1.53	**
P46019	PHKA2	729	S	1.75	*
Q9ULV1	FZD4	418	S	−3.06	***
Q05469	LIPE	853	S	−1.69	***
P10909-4	CLU	259	S	−2.97	**
H7C1J8	HNRNPA3	95	S	−1.72	**
B4DDC6	PTGES3	46	S	−2.94	*

Protein ID refers to the UniProt accession. Modified residues: S (serine), T (threonine); Y (tyrosine). Significance: *** (*P*<0.001). ** (*P*<0.01). * (*P*<0.05).

Glycogen synthase kinase-3α, termed as GSK3A, which was originally found to be associated with Alzheimer’s disease [37]. Mouse model shows that targeted disruption of GSK3A results in a decrease in sperm motility and causes male infertility [38]. In human sperm, a previous study showed that the activity of GSK3A is negatively correlated with sperm motility [39]. A recent phosphoproteomics study of human sperm also indicated that the phosphorylation level of GSK3A itself is involved in regulating sperm motility [40]. These findings provided experimental evidences that GSK3A may be the key kinase that responsible for the decrease in sperm motility during cryopreservation. The specific substrate sites of GSK3A in human sperm are not well-studied previously. However, for the first time, we provided a list of potential substrates that may help us understand the regulation of sperm motility via GSK3A. Among the substrate genes of GSK3A, three genes (GSK3A, PRKACA, and LIPE) are annotated to be directly associated with sperm motility. However, the DE site of LIPE was down-regulated. Besides the two up-regulated sites of GSK3A (autophosphorylation sites), there are also two up-regulated sites for PRKACA. PRKACA is cAMP-dependent protein kinase catalytic subunit α. It is also well known that PRKACA and PRKACA-mediated protein phosphorylations are crucial for the regulation of regulation of sperm motility and capacitation (hyperactivated motility) [41]. As a potential substrate of GSK3A, it has the most number of interacting proteins (including eight proteins). Thus, it may also play as an important mediator in the following kinase cascades of GSK3A signaling and as well as regulating the sperm motility. Combining the above results, we proposed a GSK3A-centric signaling pathway for explaining the molecular mechanism of cryodamage during sperm crypreservation (Figure 5C). In brief, the process of sperm freezing may first abnormally activate GSK3A. Then, the phosphrylation cascades transducted directly by GSK3A or indirectly by GSK3A interacting proteins are subsequently dysregulated and finally result in the decreasing sperm motility. Although other factors or pathways may also contribute to the impairment of sperm motility, we proposed a highly reliable interpretation from the perspective of phosphorylation signaling, with phosphoproteomics, bioinformatics, and experimental evidences.

### Verification of the activity of GSK3A by label-free phosphoproteomics

**Figure 6:**
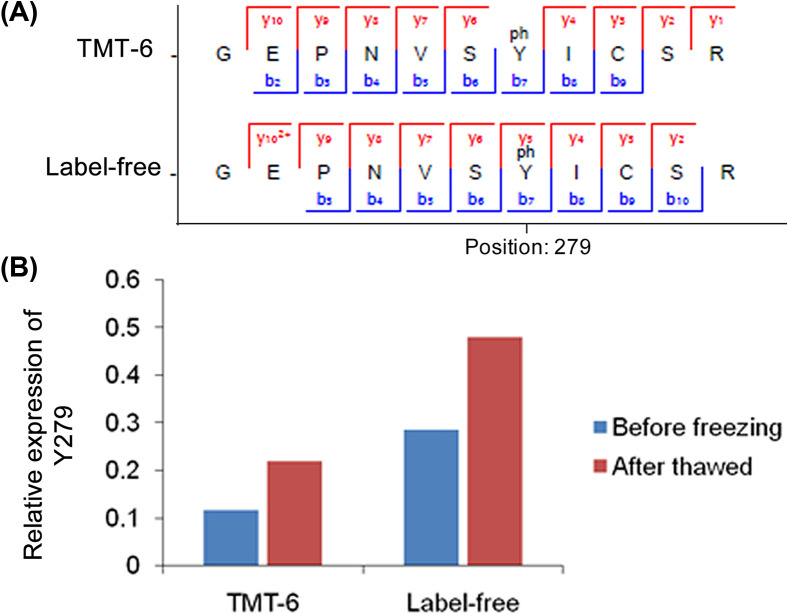
Verification of the expression of Y279 (**A**) Comarison of the fragmented ions of Y279 peptides between TMT-6 and label-free phosphoproteomics experiments. (**B**) Compariosn of the expression change of the phosphorylation level of Y279.

As mentioned above, the Y279 residue of GSK3A determines its phosphorylation activity. The phosphoprotoemic data based on TMT labeling showed that the phosphorylation level of Y279 is up-regulated with a fold change of 1.88 after sperm cryopreservation. To further verify the existence and expression of Y279, we also performed a label free-based quantitative phosphoproteomics analysis using a small size of independent samples. Comparing the spectra of Y279, both TMT and label-free experiments identify most of the fragment ions, providing strong evidences for the existence of Y279 (Figure 6A). Additionally, the label-free results also verify that Y279 site is up-regulated in the thawed sperm samples with a fold change of 1.67 (Figure 6B). Since Y279 is also the autophosphorylation site of GSK3A, this result also verifies the expression change of GSK3A substrate site.

## Conclusions

In summary, we first verified that the decreasing sperm motility is the main phenotype of cyroinjury during sperm cryopreservation. Most of previous proteomics studies used the fresh sperm as the control group for the identification of potential proteins that are responsible for the cryodamage. We thus optimized the experimental design to treat equilibrated sperm sample with the addition of cryoprotectant as the control group. Focused on the expression change of phosphorylated sites during sperm cryopreservation, we constructed a quantitative phosphoproteome of sperm proteins based on phosphopeptides enrichment and TMT labeling. A total of 3107 phosphorylated sites are identified and 848 of them are found to be significantly DE. Bioinformatics analysis showed that the corresponding genes of these DE sites are highly associated with sperm motility, providing a connection between the molecular basis and the phenotype of cryodamage. We further performed kinase enrichment analysis and successfully identified GSK3A as the key kinase that may play an important role in the regulation of sperm motility. Finally, we constructed a GSK3A centric network that could help us better understand the molecular mechanism of cryodamage in sperm motility. The presented phosphoproteome and functional associations also provide abundant research resources for us to learn the regulation of sperm functions.

Previous studies already showed that GSK3A is functionally associated with sperm motility. Here, we showed that GSK3A is abnormally activated during sperm cryopreservation. We also verified the expression of the active site of GSK3A using label-free phosphoproteomics. However, the substrates of GSK3A and following signaling cascades are still needed to be clarified in the future. Moreover, there may be other regulators and signaling pathways that involved in this process. Nevertheless, we provide strong evidences that GSK3A and its phosphorylated substrates are highly involved in regulating sperm motility. We also believe that these finding may help us to optimize the cryoprotectant for sperm cryopreservation.

## Supplementary Material

Supplementary Figures S1-S5

Supplementary Data S1-S3

## Data Availability

The data are provided as supplementary files.

## Abbreviations

DE: differentially expressed
FDR: false discovery rate
GO: gene ontology
GSK3A: glycogen synthase kinase-3α
LC-MS/MS: liquid chromatography-tandem mass spectrometry
MP: Mammalian Phenotype
PRKACA: cAMP-dependent protein kinase catalytic subunit α
PTM: post-translational modification
TMT: Tandem Mass Tag
