# Supplementary material for: Quantitative phosphoproteomics reveals GSK3A substrate network is involved in the cryodamage of sperm motility
Source: Biosci Rep. 2021 Oct 12;41(10):BSR20211326. doi: 10.1042/BSR20211326 (PMC8521533; doi:10.1042/BSR20211326)
Supplement: Supplementary Figures S1-S5 [file BSR-2021-1326_supp.pdf]

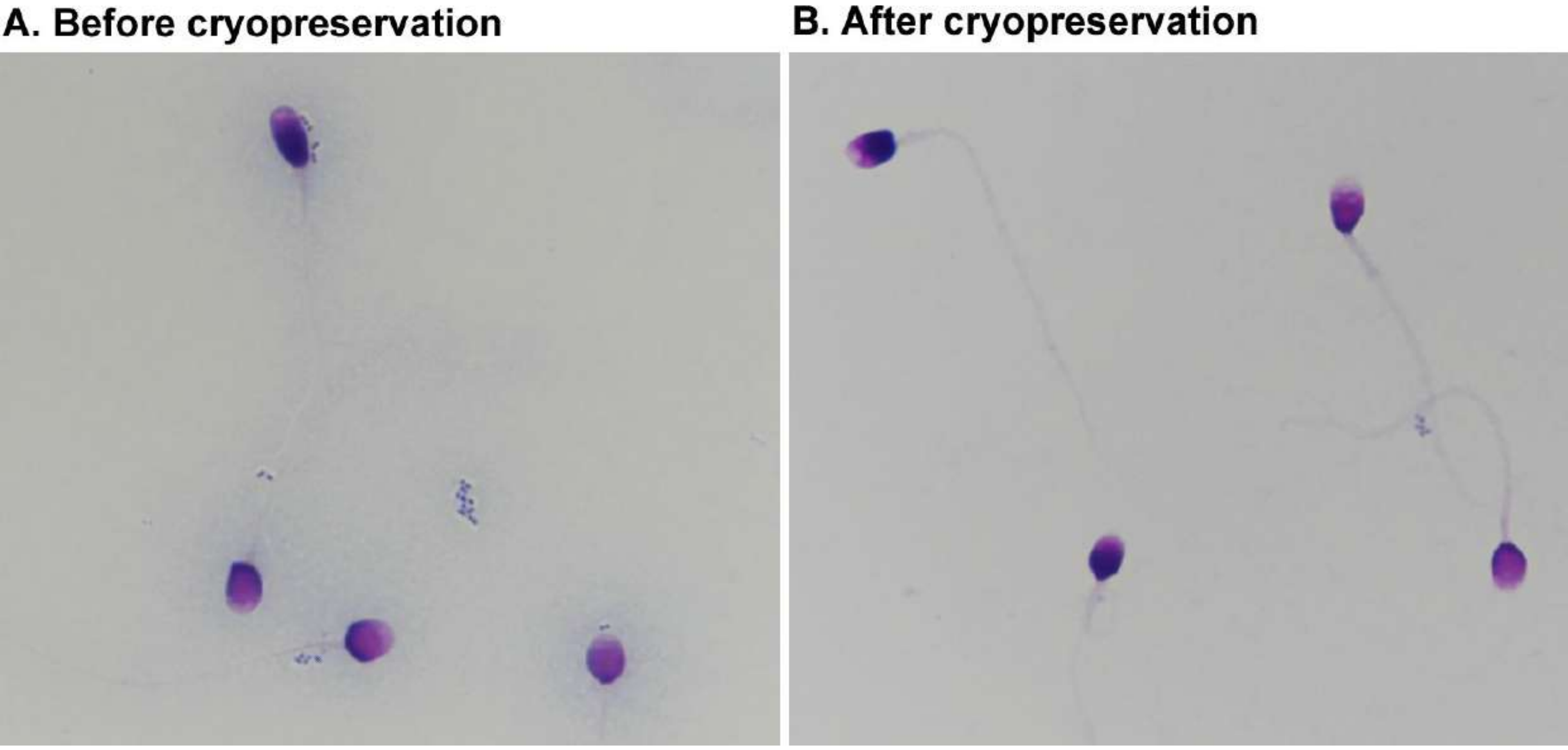
A. Before cryopreservation
B. After cryopreservation

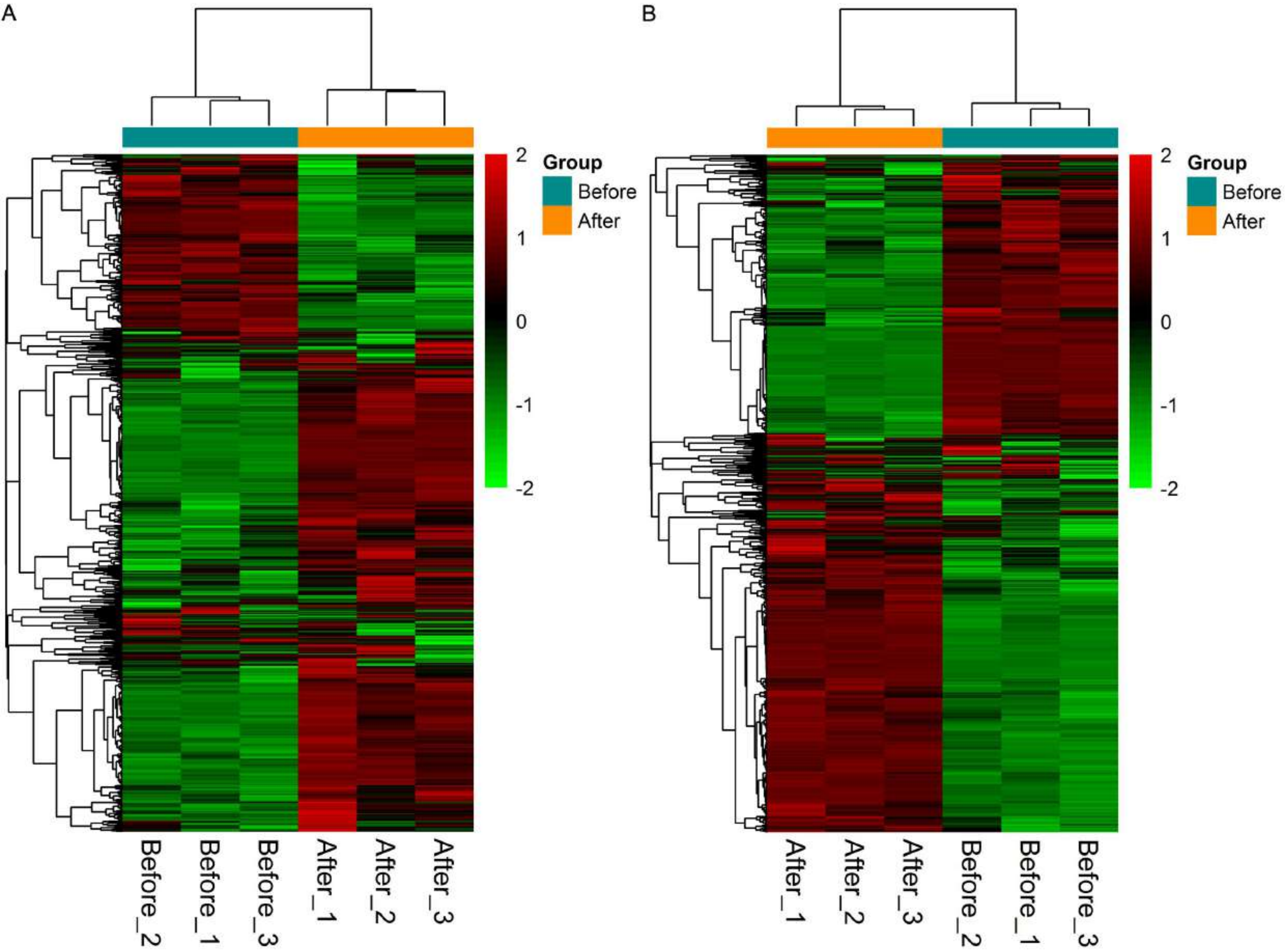
A
B
Group
Before
After
2
1
0
-1
-2
Before_2
Before_1
Before_3
After_1
After_2
After_3

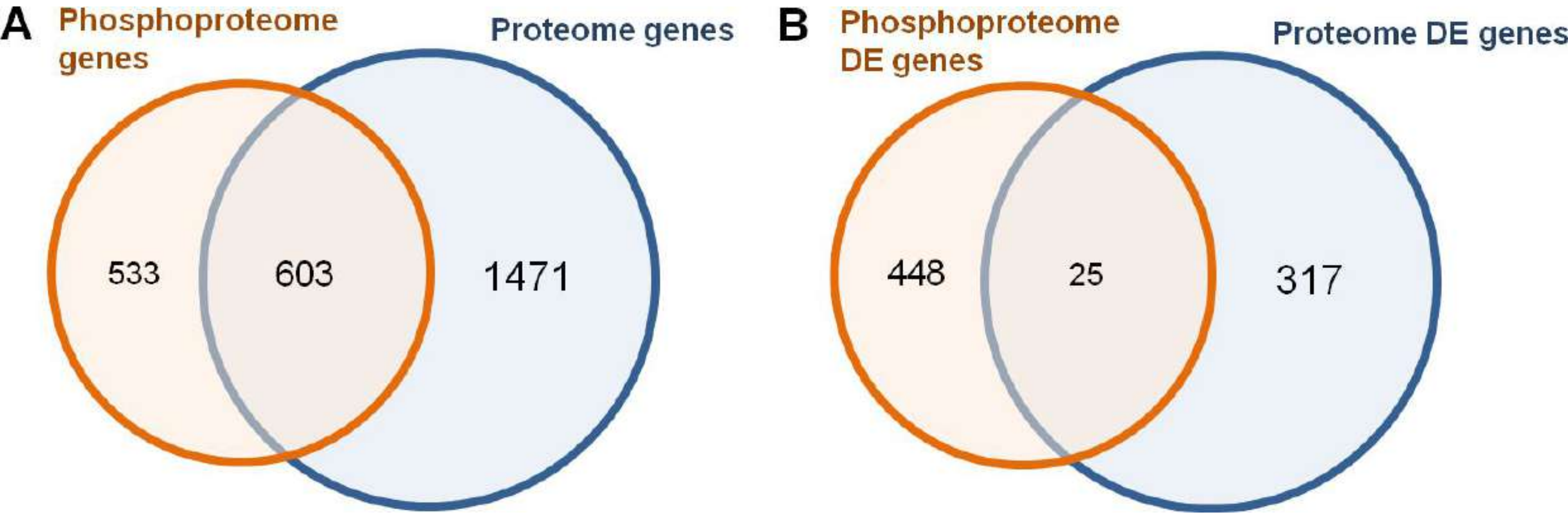
A
Phosphoproteome genes
Proteome genes
533
603
1471
B
Phosphoproteome DE genes
Proteome DE genes
448
25
317

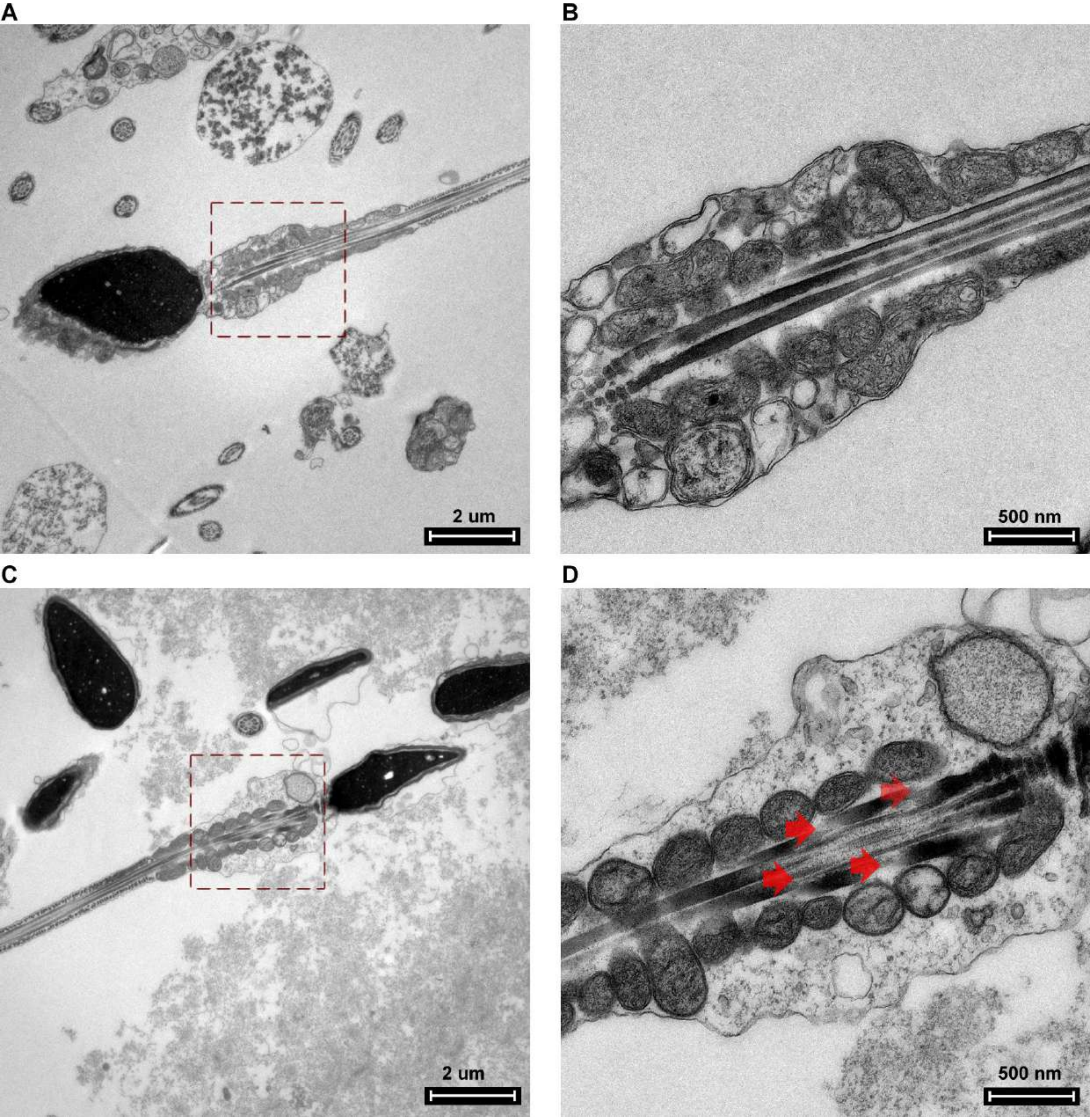
A
B
C
D
2 um
500 nm
2 um
500 nm

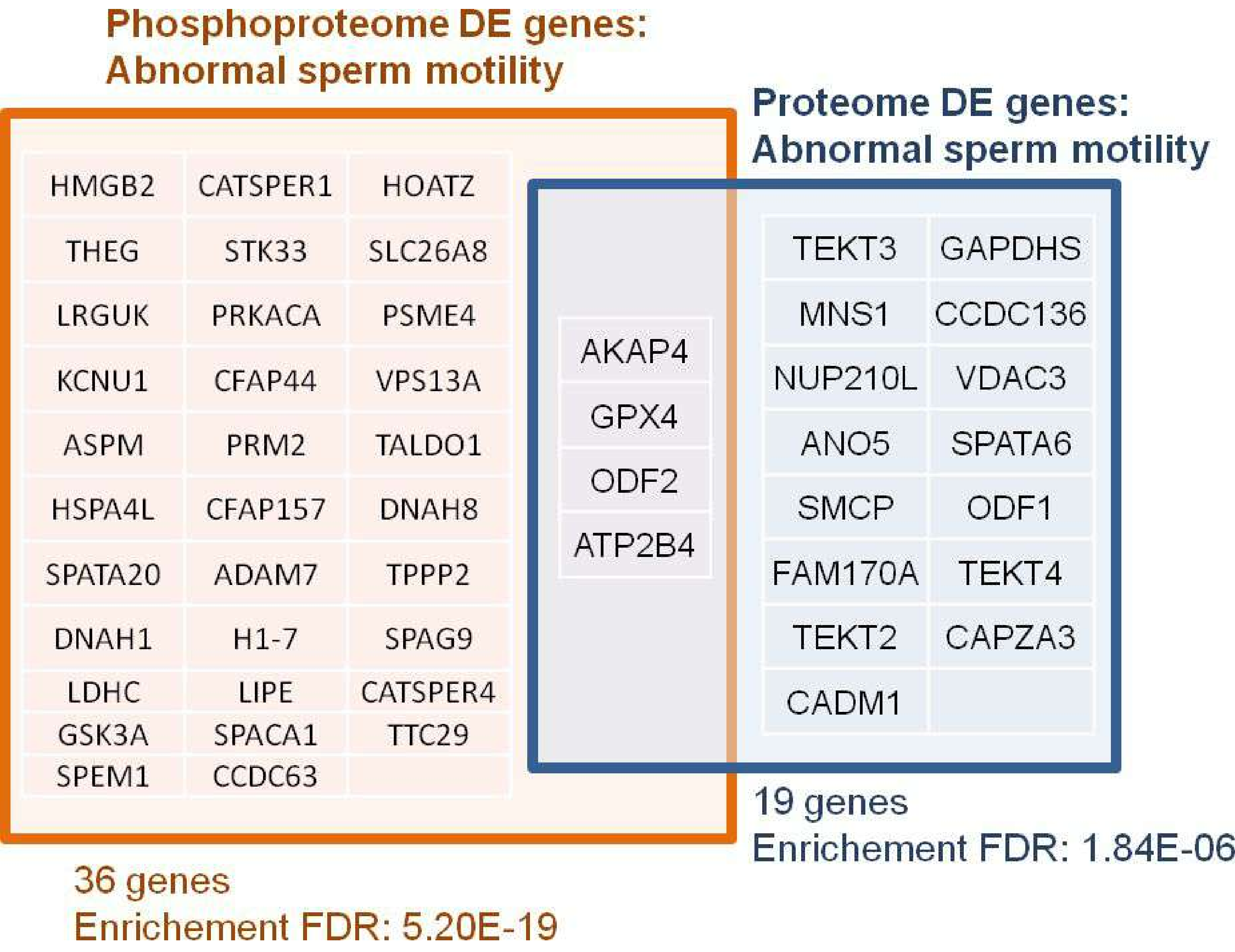
Phosphoproteome DE genes:
Abnormal sperm motility
Proteome DE genes:
Abnormal sperm motility
HMGB2
CATSPER1
HOATZ
THEG
STK33
SLC26A8
LRGUK
PRKACA
PSME4
KCNU1
CFAP44
VPS13A
ASPM
PRM2
TALDO1
HSPA4L
CFAP157
DNAH8
SPATA20
ADAM7
TPPP2
DNAH1
H1-7
SPAG9
LDHC
LIPE
CATSPER4
GSK3A
SPACA1
TTC29
SPEM1
CCDC63
AKAP4
GPX4
ODF2
ATP2B4
TEKT3
GAPDHS
MNS1
CCDC136
NUP210L
VDAC3
ANO5
SPATA6
SMCP
ODF1
FAM170A
TEKT4
TEKT2
CAPZA3
CADM1
19 genes
Enrichement FDR: 1.84E-06
36 genes
Enrichement FDR: 5.20E-19
